# Supplementary material for: Cloud BioLinux: pre-configured and on-demand bioinformatics computing for the genomics community
Source: BMC Bioinformatics. 2012 Mar 19;13:42. doi: 10.1186/1471-2105-13-42 (PMC3372431; doi:10.1186/1471-2105-13-42)
Supplement: Additional file 1 — Supplementary 1 Cloud BioLinux software documentation in the form of a mini, self-contained website. Users need to download and uncompress the .zip file, and open through a web browser the "index.html" file available on the main directory. (ZIP 1823 kb). [file 1471-2105-13-42-S1.ZIP › Cloud-BioLinux-Package-Documentation/docs/chimeraslayer.html]

Bio-Linux Software Documentation Pages

Back to search form

## chimeraslayer

|  |  |
| --- | --- |
| Name | chimeraslayer |
| Description | **chimeraslayer** is one of the programs released in the microbiomeutil package from the Broad Institute. chimeraslayer is a chimeric sequence detection utility that can work with near-full length Sanger sequences and shorter 454-FLX sequences (~500 bp).  More details about chimeraslayer can be found via the microbiomeutil website. |
| Homepage | http://microbiomeutils.sourceforge.net |
| Remote Documentation | http://microbiomeutil.sourceforge.net/#A\_CS |
